# Renal Expression of FGF23 in Progressive Renal Disease of Diabetes and the Effect of Ace Inhibitor

**DOI:** 10.1371/journal.pone.0070775

**Published:** 2013-08-14

**Authors:** Cristina Zanchi, Monica Locatelli, Ariela Benigni, Daniela Corna, Susanna Tomasoni, Daniela Rottoli, Flavio Gaspari, Giuseppe Remuzzi, Carlamaria Zoja

**Affiliations:** 1 IRCCS – Istituto di Ricerche Farmacologiche Mario Negri, Centro Anna Maria Astori, Science and Technology Park Kilometro Rosso, Bergamo, Italy; 2 IRCCS – Istituto di Ricerche Farmacologiche Mario Negri, Clinical Research Center for Rare Diseases Aldo e Cele Daccò, Ranica, Bergamo, Italy; 3 Unit of Nephrology and Dialysis, Azienda Ospedaliera Papa Giovanni XXIII, Bergamo, Italy; Fondazione IRCCS Ospedale Maggiore Policlinico & Fondazione D'Amico per la Ricerca sulle Malattie Renali, Italy

## Abstract

Fibroblast growth factor 23 (FGF23) is a phosphaturic hormone mainly produced by bone that acts in the kidney through FGF receptors and Klotho. Here we investigated whether the kidney was an additional source of FGF23 during renal disease using a model of type 2 diabetic nephropathy. Renal expression of FGF23 and Klotho was assessed in Zucker diabetic fatty (ZDF) and control lean rats at 2, 4, 6, 8 months of age. To evaluate whether the renoprotective effect of angiotensin converting enzyme (ACE) inhibitor in this model was associated with changes in FGF23 and Klotho, ZDF rats received ramipril from 4, when proteinuric, to 8 months of age. FGF23 mRNA was not detectable in the kidney of lean rats, nor of ZDF rats at 2 months of age. FGF23 became measurable in the kidney of diabetic rats at 4 months and significantly increased thereafter. FGF23 protein localized in proximal and distal tubules. Renal Klotho mRNA and protein decreased during time in ZDF rats. As renal disease progressed, serum phosphate levels increased in parallel with decline of fractional phosphorus excretion. Ramipril limited proteinuria and renal injury, attenuated renal FGF23 upregulation and ameliorated Klotho expression. Ramipril normalized serum phosphate levels and tended to increase fractional phosphorus excretion. These data indicate that during progressive renal disease the kidney is a site of FGF23 production which is limited by ACE inhibition. Interfering pharmacologically with the delicate balance of FGF23 and phosphorus in diabetes may have implications in clinics.

## Introduction

Fibroblast growth factor (FGF) 23 is a phosphaturic hormone produced in response to an increase in phosphorus load or high levels of calcitriol or parathyroid hormone [1]–[3]. FGF23 acts by inducing renal phosphate excretion by kidney proximal tubular cells through reduction of the expression of type 2a and 2c sodium phosphate co-transporters (NaPi-2a and NaPi-2c) [4]. FGF23 also suppresses the production of vitamin D's active form (1,25-dihydroxyvitamin D) in the kidney by inhibiting the synthetic enzyme 1α-hydroxylase, thereby acting as counter-regulatory hormone for vitamin D [1], [5]. The reduction in circulating 1,25-dihydroxyvitamin D levels by FGF23 contributes to cause negative phosphate balance through limiting phosphate absorption from the intestine [6]. FGF23 exerts its intrarenal biological function by binding to cognate FGF receptors (FGFRs) requiring the presence of Klotho, a transmembrane protein highly expressed in the kidney, as a co-receptor [7]. The site of synthesis of FGF23 is primarily the bone tissue, more specifically osteocytes and osteoblasts, although FGF23 is also espressed by brain, thymus, liver, spleen and heart [8]–[10]. Since the kidney is an important target of FGF23, and the circulating levels of FGF23 have been found to increase in association with disease progression and cardiovascular events in chronic kidney disease and diabetic nephropathy [1], [2], [11], we wondered whether the kidney could be a source of FGF23 during the development of renal disease. Few data are so far available showing that renal tissue expressed FGF23 at very low level, if any, in normal conditions [8], [12], [13] and in uremic rats [8], [13], [14].

We took advantage of the Zucker diabetic fatty (ZDF) rat model of human type 2 diabetic nephropathy characterized by obesity, hyperlipidemia, insulin resistance, progressive renal injury and cardiac abnormalities [15]–[18] and evaluated the expression of FGF23 in the kidney during the course of the disease. We also investigated whether renoprotective effects of ACE inhibitor in this model [19] were associated with modulation of renal FGF23 and Klotho expression.

## Methods

### Experimental animals

Male ZDF rats (ZDF/Gmi-*fa/fa*) and aged-matched non-diabetic lean rats (ZDF/Gmi-*fa/+*) (Charles River Laboratories Italia S.r.l., Calco, Italy) were kept on a 12-hour light/dark cycle with free access to water. ZDF rats were maintained on Purina 5008 rat chow to accelerate onset of diabetes. Phosphate content of Purina 5008 diet was similar to that of rat standard diet. Animal care and treatment were conducted according with institutional guidelines in compliance with national (Decreto Legislativo n.116, Gazzetta Ufficiale suppl 40, 18 febbraio 1992, Circolare n.8, Gazzetta Ufficiale 14 luglio 1994) and international laws and policies (EEC Council Directive 86/609, OJL358–1, December 1987; Guide for the Care and Use of Laboratory Animals, US National Research Council, 1996). [19]. Animal studies were approved by the Institutional Animal Care and Use Committee of IRCCS – Istituto di Ricerche Farmacologiche Mario Negri. ZDF (n = 30) and lean (n = 20) rats were studied from 2 months of age. Starting from 4 months ZDF rats received daily vehicle or ramipril (1 mg/kg in the drinking water) [19] until 6 or 8 months. Rats were sacrificed at 2, 4, 6, 8 months of age (n = 5 rats/each group).

### Laboratory parameters

Blood glucose was assessed with reflectance meter (OneTouch UltraEasy, LifeScan, Milan, Italy). Serum cholesterol, triglycerides, BUN, serum and urinary creatinine were measured by a Cobas Mira autoanalyzer (Roche Diagnostic Systems, Basel, Switzerland). Serum FGF23 levels were measured by rat FGF23 ELISA kit (Kainos Laboratories, Inc, Tokyo, Japan). Serum and urinary phosphorus were assessed by autoanalyzer (CX5, Beckman Instruments Inc., Fullerton, CA, USA). Fractional excretion of phosphorus was calculated using the formula: [urine phosphate x serum creatinine] ×100/[serum phosphate x urine creatinine]. The tubular maximum reabsorption of phosphorus per glomerular filtration rate (TmP/GFR) was calculated using the formula: serum phosphate -[(urine phosphate x serum creatinine)/urine creatinine] (mg/dl). Proteinuria was measured by the blue Coomassie method using a Cobas Mira autoanalyzer. Systolic blood pressure was evaluated with a computerized tail-cuff system in conscious rats.

### Real time quantitative (q) PCR and reverse-transcriptase (RT)-PCR

Total RNA was extracted from whole kidney tissue using TRIzol reagent (Life Technologies, Monza, Italy). Purified RNA (2 µg) was reverse transcribed. qPCR was performed on 7300 Real-Time PCR System (Life Technologies) using the following TaqMan Gene Expression Assays: Rn00590709_m1 for FGF23, Rn01402172_m1 for Klotho, Rn00564677_m1 for NaPi-2a and Rn00595128_m1 for NaPi-2c. Rat β-actin endogenous control (VIC/MGB probe) was used as reference gene. The cDNA content of each sample was calculated using the ΔΔCt technique. RT-PCR for FGF23 mRNA expression was performed in Perkin-Elmer 2700 thermal cycler (Life Technologies) by 35 cycles of denaturation (94°C, 30 sec), annealing (60°C, 1 min) and extension (72°C, 1 min). The following primers were used: forward 5′-CCCATCAGACTATCTACAGTGCCC-3′; reverse 5′-GCTTCGGTGACAGGTAGACGTC-3′. The amplification products were detected on 2% agarose gel and their identity confirmed by sequencing.

### Renal histology

Paraffin-embedded kidney sections (3 µm) were stained with hematoxylin/eosin and periodic-acid Schiff reagent. The extent of glomerular damage was expressed as percentage of sclerotic glomeruli. At least 100 glomeruli were examined for each animal. Tubular changes (atrophy, dilatation, hyaline casts) were graded from 0 to 4 (0, no changes; 1, changes affecting 25% of the sample; 2, changes affecting >25–50% of the sample; 3, changes affecting >50–75% of the sample; 4, changes affecting >75–100% of the sample). Kidney biopsies were analyzed by the same pathologist, who was unaware of the nature of the experimental groups.

### Immunohistochemistry

Mouse monoclonal anti-rat antibody (1∶100, Chemicon, Temecula, CA) was used for the detection of monocyte/macrophage ED-1 surface antigen by alkaline phosphatase-Fast Red technique on paraffin-embedded sections. ED-1 positive cells were counted in 30 randomly selected interstitial high-power microscopic fields (HPF, ×400) for each animal. FGF23, Klotho and NaPi-2a proteins were detected by immunoperoxidase 3,3-diaminobenzidine technique with Vectastain ABC kit (Vector Laboratories, Burlingame, CA) on paraffin-embedded sections using as primary antibodies: rabbit anti-mouse FGF23 antibody (1∶100, Santa Cruz Biotechnology, Santa Cruz, CA, USA) detecting FGF23 of both mouse and rat origin or goat anti-human FGF23 antibody (1∶200, Abcam, Cambridge, UK) also detecting rat FGF23 protein [20], goat anti-human Klotho antibody (1∶100, Santa Cruz Biotechnology), rabbit anti-human NaPi-2a antibody (1∶50, Alpha Diagnostic, San Antonio, TX, USA), followed by biotinylated antibodies. Nuclei were counterstained with Harris hematoxylin. Negative controls were obtained by omitting the primary antibody on adjacent sections.

### Western blot analysis

Frozen kidney tissues were homogenized in lysis buffer (50 mM Tris-HCl (pH 8), 150 mM NaCl, 1% Triton X-100, 0.5% Sodium deoxycholate, 0.1% SDS) containing the Halt protease inhibitor cocktail (Thermo Scientific, Rockford, IL, USA). Protein concentration was determined using the bicinchoninic acid assay (Thermo Scientific) following the manufacturer's instructions. The samples (50 µg) were resolved on 10% SDS-polyacrylamide gel and transferred to nitrocellulose membranes. After blocking, the blots were incubated with rabbit anti-human NaPi-2a antibody (1∶200, Alpha Diagnostic) or mouse monoclonal anti-α-tubulin antibody (1∶4000; Sigma-Aldrich, St Louis, MO, USA) overnight at 4°C and with an appropriate secondary antibody linked to horseradish peroxidase (Sigma-Aldrich). Protein bands were detected by Supersignal chemiluminescent substrate (Thermo Scientific) and quantified using NIH Image J Software. The amount of NaPi-2a protein was calculated relative to the level of α-tubulin.

### Statistical analysis

Results are mean ± SEM. Serum triglycerides and proteinuria data that are not normally distributed are expressed as median and interquartile range (IQR). Data were analyzed using ANOVA coupled with Bonferroni post hoc analysis or non-parametric Mann-Whitney or Kruskal-Wallis test for multiple comparisons, as appropriate. The degree of association between renal FGF23 expression and renal damage markers was assessed by calculating non parametric Spearman's rho coefficient. Statistical significance level was defined as *p*<0.05.

## Results

### Characteristics of the type 2 diabetes model of ZDF rats

**Figure 1:**
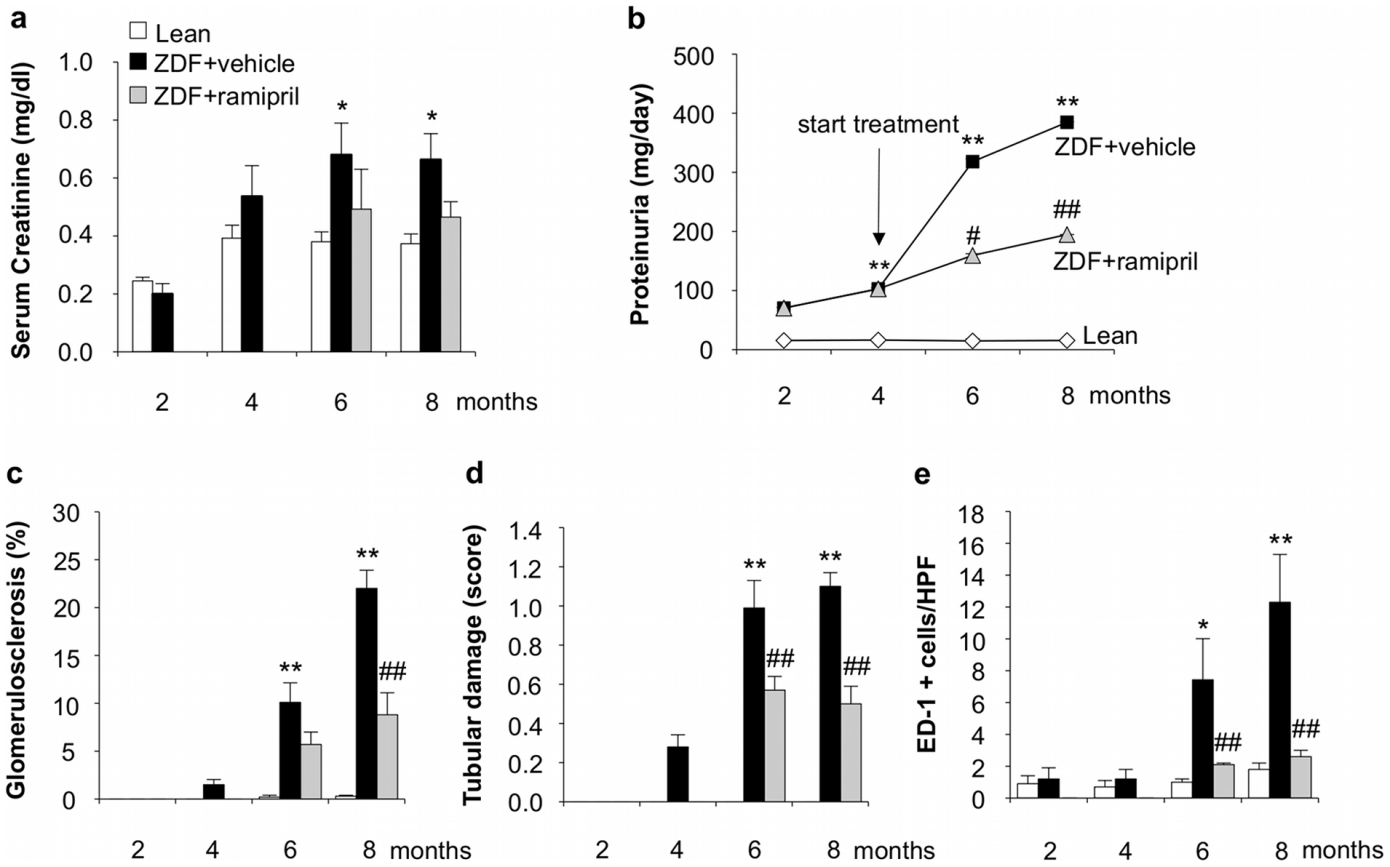
Renal functional parameters and structural changes in ZDF rats, and effect of ACE inhibitor therapy. (**a**) Serum creatinine, (**b**) proteinuria, (**c**) glomerulosclerosis, (**d**) tubular damage and (**e**) interstitial accumulation of ED-1 positive monocytes/macrophages were evaluated during time in lean rats, and in ZDF rats receiving vehicle or ramipril from 4 to 8 months of age (n = 5 rats each group/time). Values are mean ± SEM or median for proteinuria. *p<0.05, **p<0.01 vs age-matched lean rats; ^#^ *p*<0.05, ^##^ *p*<0.01 vs ZDF+vehicle.

**Figure 2:**
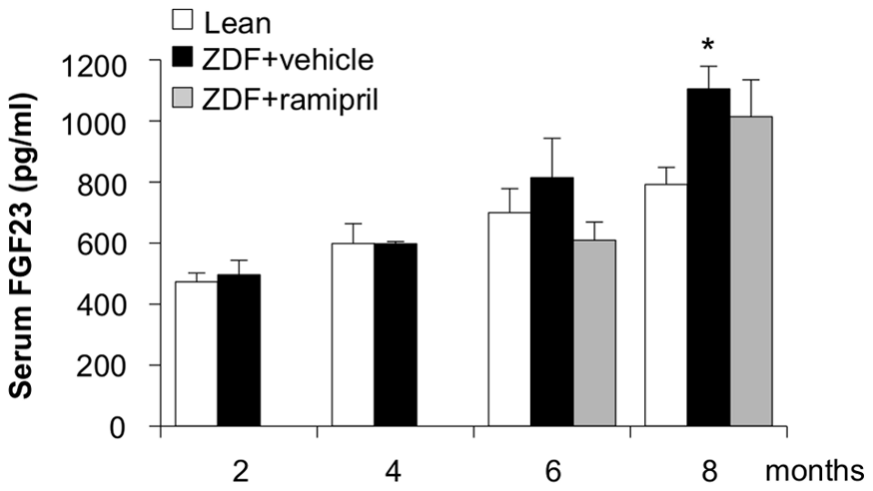
Time course of serum FGF23 levels in lean and ZDF rats. Serum FGF23 was measured in lean rats, and in ZDF rats receiving vehicle or ramipril from 4 to 8 months of age (n = 5 rats each group/time). Values are mean ± SEM. *p<0.05 vs lean rats at 8 months.

Food intake was significantly higher in ZDF rats with respect to lean rats during the whole study period (2 months: 24±2 vs 17±1 g/day, *p*<0.05; 4 months: 30±1 vs 16±1 g/day, *p*<0.01; 6 months: 29±2 vs 17±1 g/day, *p*<0.01; 8 months: 27±1 vs 17±1 g/day, *p*<0.01). ZDF rats exhibited hyperglycemia, abnormal lipid profile, mild hypertension and increased BUN and serum creatinine levels with respect to lean rats (Table 1 and Fig. 1a), in line with previous observations [19]. As shown in Fig. 1b, proteinuria progressively increased during time reaching at 8 months of age 385 mg/day (median; IQR: 330–531, vs lean rats: 16 (14–20) mg/day). By 6 months, ZDF rats developed glomerulosclerosis that became more severe at 8 months affecting on average 22±2% of glomeruli (Fig. 1c). Tubular damage consisting of atrophy, dilatation and hyaline casts, and interstitial inflammation, evaluated as accumulation of ED1-positive monocytes/macrophages, were also present and worsened during the disease (Fig. 1d and e). The time course of serum levels of FGF23 is reported in Fig. 2. Levels were significantly (*p*<0.05) increased in ZDF rats with respect to lean rats at 8 months.

**Table 1:** Systemic and laboratory parameters in lean and ZDF rats at 8 months of age.

Groups	Body weight *(g)*	Food Intake *(g/day)*	Blood Glucose *(mg/dl)*	Serum Cholesterol *(mg/dl)*	Serum triglycerides *(mg/dl)*	Blood pressure *(mmHg)*	BUN *(mg/dl)*
Lean	444±9	17±1	110±2	115±5	146 (124–204)	139±3	27±2
ZDF+vehicle	391±14*	27±1**	506±22**	369±25**	1420 (929–1592) **	145±3	42±5*
ZDF+ramipril	387±8**	29±3**	513±9**	284±15**^,#^	824 (775–932)**	121±5*^,##^	33±4

Values are mean ± SEM or median (IQR). *p<0.05, **p<0.01 vs lean; ^#^ *p*<0.05, ^##^ *p*<0.01 vs ZDF+vehicle.

### FGF23 and Klotho expression in the kidney of ZDF rats

**Figure 3:**
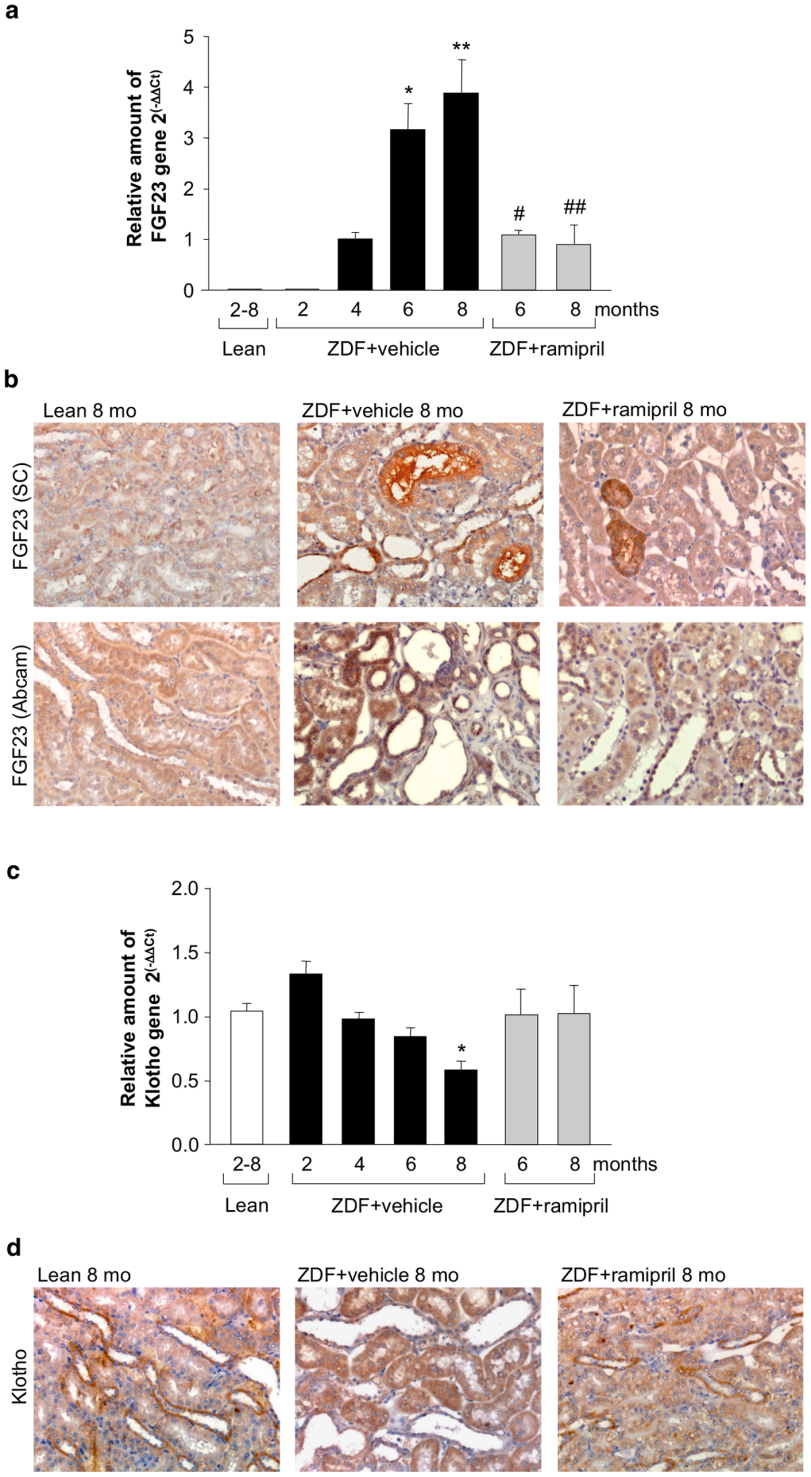
Renal expression of FGF23 and Klotho during the course of the disease of ZDF rats, and modulation by ACE inhibitor therapy. (**a**) FGF23 mRNA expression evaluated in kidney tissue from lean and ZDF rats at different ages, and effect of ACE inhibitor treatment (n = 5 rats each group/time). FGF23 mRNA was not detectable in the kidneys of lean rats at all times period nor in ZDF rats at 2 months of age; it was measurable in the kidney of 4-month old ZDF rats. Relative expression values were calculated by the ΔΔCt method using the cDNA expression of ZDF at 4 months as reference. Data are mean ± SEM. *p<0.05, **p<0.01 vs ZDF rats at 4 months; ^#^ *p*<0.05, ^##^ *p*<0.01 vs ZDF+vehicle rats at corresponding age. (**b**) Representative images of FGF23 staining in the kidney of lean rats and ZDF rats receiving vehicle or ramipril at 8 months of age. FGF23 expression was detected by immunoperoxidase technique using two different anti-FGF23 antibodies (from Santa Cruz Biotechnology, SC, or Abcam, see Methods). Magnification, ×400. (**c**) Time course of Klotho mRNA expression in the kidney of lean rats and ZDF rats given vehicle or ramipril (n = 5 rats each group/time). Relative expression values in each group were calculated vs lean rats of corresponding age. Data are mean ± SEM. *p<0.05 vs lean rats. (**d**) Representative images of Klotho staining by immunohistochemistry in the kidney of lean rats and ZDF rats treated with vehicle or ramipril, at 8 months of age. Magnification, ×400.

The expression of FGF23 mRNA was evaluated in the kidney of lean and ZDF rats at 2, 4, 6, 8 months of age by either qPCR (Fig. 3a) or RT-PCR (Fig. S1). FGF23 mRNA was not detectable in the kidney of lean rats at all the time points nor in the kidney of ZDF rats at 2 months, but became measurable at 4 months and further increased thereafter. By immunohistochemistry, using two different anti-FGF23 antibodies, no FGF23 signal was found in lean or ZDF rats at 2 months of age (not shown), whereas focal staining of FGF23 was present in both proximal and distal tubular profiles at a more advanced phase of the disease (Fig. 3b). Concomitant to renal FGF23 overexpression, renal Klotho mRNA levels and protein staining, observed in distal tubules, decreased during time in ZDF rats with respect to lean rats (Fig. 3c and d).

### Renal expression of sodium phosphate co-transporters and phosphate metabolism in ZDF rats

**Figure 4:**
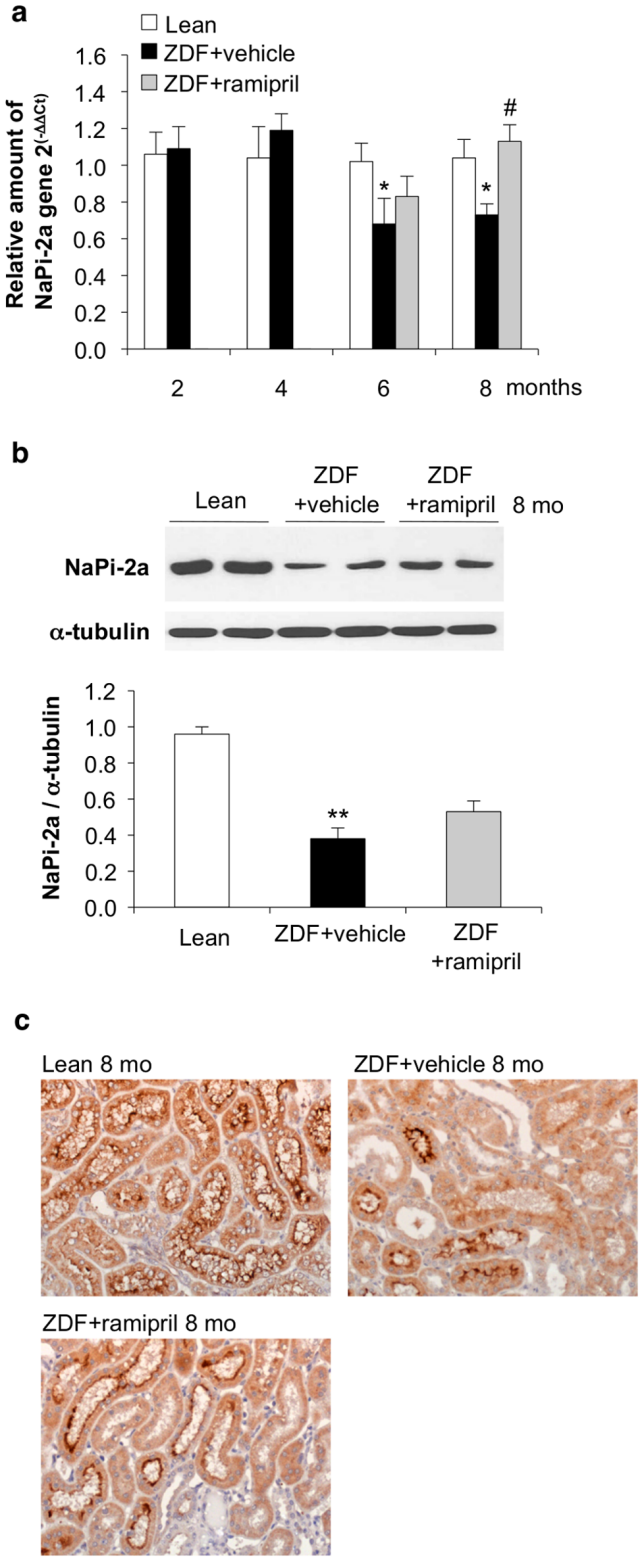
Renal expression of sodium phosphate (NaPi)-2a co-transporter in ZDF rats, and effect of ramipril. (**a**) mRNA expression of NaPi-2a co-transporter evaluated in the kidney of lean rats, and ZDF rats treated with vehicle or ramipril from 4 to 8 months of age (n = 5 rats each group/time). Relative expression values were calculated versus lean rats of corresponding age. Data are mean ± SEM. *p<0.05 vs age-matched lean rats; ^#^ *p*<0.01 vs ZDF+vehicle. (**b**) Western blot analysis for NaPi-2a protein expression, relative to α-tubulin, in lean rats and ZDF rats treated with vehicle or ramipril, at 8 months of age (n = 5 rats each group). Data are mean ± SEM. **p<0.01 vs lean rats. (**c**) Representative images of NaPi-2a staining by immunohistochemistry in the kidney of lean rats, and ZDF rats receiving vehicle or ramipril, at 8 months of age. Magnification, ×400.

Since FGF23 down-regulates the expression of sodium phosphate co-transporters in the proximal tubules, thereby diminishing phosphate reabsorption and increasing urinary phosphate excretion [1]–[3], we evaluated the expression of NaPi-2a and NaPi-2c in the kidney of ZDF rats during the course of the disease. NaPi-2a is the protein mainly responsible for phosphate reabsorption in the kidney [21]. NaPi-2a mRNA was normally expressed at 4 months of age but significantly reduced at 6 and 8 months in diabetic rats with respect to controls (Fig. 4a). Western blot analysis showed a significant decrease of NaPi-2a protein expression in the kidney of ZDF rats at 8 months of age versus lean rats (Fig. 4b). These results were confirmed by immunohistochemistry analysis showing a reduced staining for NaPi-2a protein at the brush border membrane of proximal tubules of ZDF rats (Fig. 4c). NaPi-2c mRNA expression was slightly but not significantly decreased in the kidney of ZDF rats at 8 months (21% reduction versus lean rats) (not shown).

**Figure 5:**
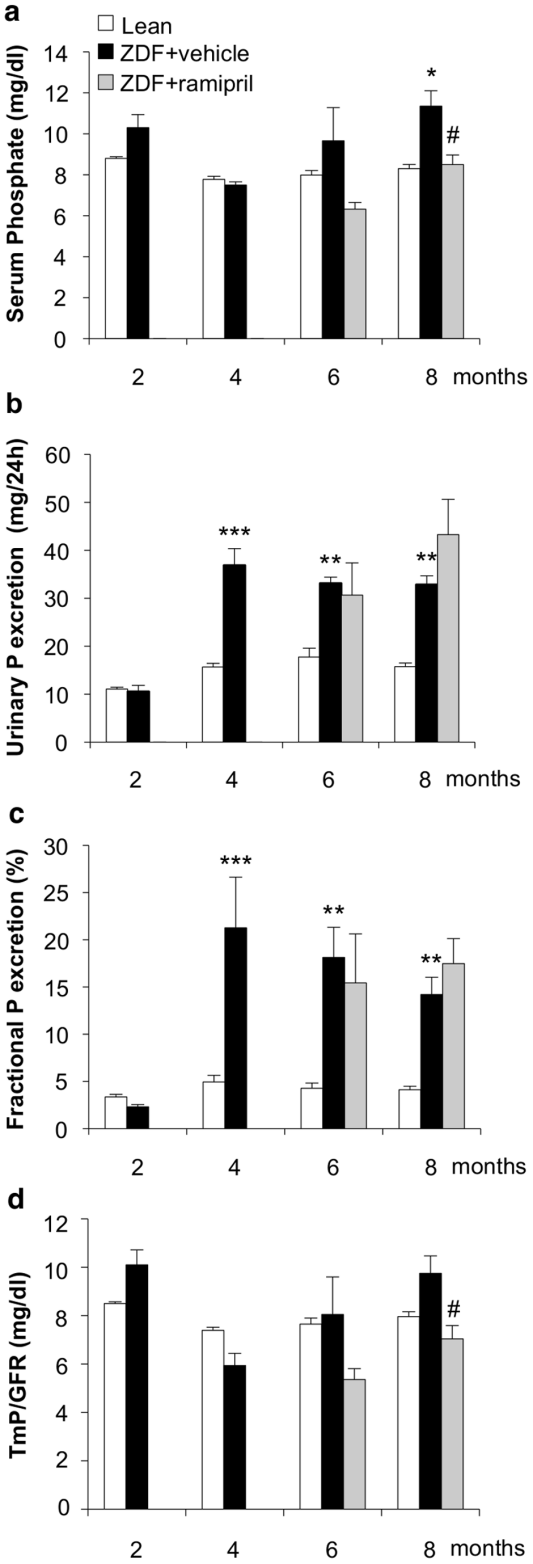
Serum phosphate levels, urinary phosphorus excretion and fractional phosphorus excretion during the course of the disease in ZDF rats, and effect of ACE inhibitor therapy. (**a**) Serum phosphate levels, (**b**) urinary phosphorus (P), (**c**) fractional P excretion and (**d**) TmP/GFR were measured over time in lean rats, and ZDF rats given vehicle or ramipril (n = 5 rats each group). Data are mean ± SEM. *p<0.05, **p<0.01, ***p<0.001 vs age-matched lean rats; ^#^ *p*<0.05 vs ZDF+vehicle.

Serum phosphate levels of ZDF rats were not different from those of lean rats until 6 months of age. A significant increase (*p*<0.05 versus lean rats) was found at 8 months (Fig. 5a). Urinary total phosphorus (Fig. 5b) and fractional excretion of phosphorus (Fig. 5c) were similar in ZDF and lean rats at 2 months. Increased levels of urinary phosphorus excretion were found in ZDF rats at 4 months, that persisted subsequently (Fig. 5b). Fractional phosphorus excretion, which was elevated at 4 months, showed a tendency to decline during the course of the disease as a consequence of renal function impairment (Fig. 5c). As shown in Fig. 5d, the ratio of the maximum rate of tubular phosphate reabsorption to the GFR (TmP/GFR) -which is referred to as the theoretical renal phosphate threshold- in ZDF rats given vehicle showed with time from 4 to 8 months a tendency to increase possibly reflecting the decrease in renal function.

### Effects of ACE inhibitor treatment in ZDF rats

To study whether renal FGF23 and Klotho expression could be modulated by ACE inhibitor, ZDF rats received ramipril from 4 months of age, a time point at which animals had developed proteinuria and signs of renal injury, up to 6 or 8 months. Accordingly to our previous study [19] ramipril limited dyslipidemia, reduced systolic blood pressure, and partially decreased BUN in ZDF rats (Table 1). Body weight and food intake were not affected by the treatment (Table 1). The time course of serum creatinine showed that ramipril prevented the worsening of renal function. Thus, serum creatinine levels at 6 and 8 months were similar to those measured at 4 months before the institution of treatment (Fig. 1a). ACE inhibitor therapy showed in this model a significant (*p*<0.01) antiproteinuric effect reaching 55% reduction of proteinuria versus vehicle at 8 months (median, 195; IQR: 133–237 mg/day) (Fig. 1b). Glomerular and tubular changes, and interstitial inflammation were also significantly attenuated (Fig. 1c, d and e). Ramipril did not affect serum FGF23 levels (Fig. 2). In the kidney of ZDF rats treated with ramipril, FGF23 mRNA expression was significantly reduced with respect to animals given vehicle, but not abrogated (Fig. 3a). In contrast, renal Klotho mRNA increased to levels near to controls (Fig. 3c). Ramipril restored renal mRNA expression of NaPi-2a at 8 months (Fig. 4a). At variance, protein expression was slightly, although not significantly increased by ACE inhibitor treatment, as shown either by Western blot or immunohistochemistry (Fig. 4b and c). Ramipril kept serum phosphate levels of ZDF rats similar to those of lean rats at 6 and 8 months, as shown in Fig. 5a. Urinary phosphorus and fractional phosphorus excretion were not significantly modified after ramipril, although a trend to increase was observed with respect to vehicle (Fig. 5b and c). Conversely, TmP/GFR showed a decrease (Fig. 5d).

### Correlation analysis

Spearman's rho rank correlation analysis showed a significant (*p*<0.0001) positive correlation between the level of renal FGF23 expression and proteinuria (rho = 0.689), glomerulosclerosis (rho = 0.823), tubular damage (rho = 0.787) and interstitial ED1-positive monocytes/macrophages (rho = 0.730). Renal FGF23 also significantly (*p*<0.01) correlated positively with fractional phosphorus excretion (rho = 0.566) and negatively with NaPi-2a co-transporter mRNA expression (rho: −0.488).

## Discussion

The present study shows that the kidney of ZDF rats, a model resembling human type 2 diabetic nephropathy, expressed FGF23 starting from the age of 4 months when rats have already significant levels of proteinuria and signs of renal injury. FGF23 mRNA expression further increased with time as diabetic disease progressed, reaching levels that were 3–4 times higher than at 4 months. To our knowledge this is the first evidence of FGF23 production by the kidney during renal disease progression. Upregulation of FGF23 mRNA resulted in the expression of the corresponding protein localized at proximal and distal tubules in focal areas of the kidney. A previous study by Mirams et al [8], [12], [13] that analyzed FGF23 mRNA expression in several human tissues showed that FGF23 was detectable in kidney tissue at low levels. Actually, the highest expression was found in bone followed by kidney medulla, liver, thyroid and kidney cortex. FGF23 bone expression was approximately 30 times higher than renal expression in normal conditions, but no data are available that compared tissues from patients with renal disease. The prevailing paradigm proposes the bone as the main tissue that senses changes in phosphate balance and produces FGF23 [3]. The present data would add the kidney as a direct sensor organ that may interfere in phosphate homeostasis via its own FGF23 production. It is noteworthy that the FGF23 co-receptor Klotho is expressed weakly in the proximal tubules -despite the fact that the FGF23 phosphaturic action is substantially exerted at this level- whereas distal tubules showed more abundant Klotho expression [22] [23]. Two mechanisms have been proposed [6]: either FGF23 acts on proximal tubules via FGFR-Klotho signaling to directly regulate NaPi co-transporters, and/or acts on the distal tubules to induce a paracrine signal to proximal tubules [24]. The putative paracrine factor is the secreted Klotho itself able to directly inhibit NaPi co-transporters and to activate several ion channels in proximal tubules [22]. The significance of the local production of FGF23 is obscure. FGF23 expression might represent an adaptive response to early injury as a mechanism by which the ZDF rat kidney could maintain phosphate homeostasis. Indeed, at 4 months, Klotho was normally expressed in the kidney allowing FGF23 phosphaturic activity to maintain serum phosphate levels similar to those of lean rats. Finding that ZDF rats exhibited a 2-fold higher urinary phosphorus excretion than lean rats could also be attributed to a 2-fold increased food intake in ZDF rats. As renal disease progressed, despite the increasing FGF23 levels, fractional phosphorus excretion decreased possibly due to a lower renal Klotho expression and a reduction in functioning nephrons, with the net result of increased serum phosphate levels. Our finding of decreased Klotho expression in the kidney of ZDF rats during overt nephropathy is in agreement with findings of decline of renal Klotho both in rodent models of chronic renal damage and patients with CKD [22], [25]. Klotho is a putative aging suppressor [26], and there is compelling evidence that its depletion is associated with oxidative stress, inflammation, accelerated aging and renal fibrosis [27], [28].

An important observation of the present study is that ramipril therapy, which limited proteinuria and renal damage in ZDF rats, effectively prevented the time-dependent increase of renal FGF23 expression, and almost normalized Klotho expression. A cross talk between the renin-angiotensin-system (RAS) and Klotho-FGF23 axis has been suggested (see for a review de Borst et al. [29]). Angiotensin II causes renal Klotho loss leading to disrupted FGF23 signaling and reduced phosphaturia [29], [30]. In a model of renal damage characterized by renal RAS activation and Klotho downregulation, the treatment with an angiotensin II type 1 receptor blocker prevented the loss of Klotho expression and ameliorated renal histology [31]. Here, ramipril by recovering renal Klotho expression allowed re-engagement of serum and residual renal FGF23 to exert phosphaturic activity, resulting in normalization of serum phosphate levels in diabetic rats. Ramipril was capable to restore the mRNA expression of NaPi-2a co-transporter to normal levels, which however, did not translate into recovery of the protein on the brush border of proximal tubules. Discrepancy between NaPi-2a co-transporter mRNA and protein expression could be attributable to post-transcriptional events that precluded a full restoration of the protein. This would explain the high level of phosphorus excretion at 8 months in ramipril-treated diabetic rats. ACE inhibitor treatment significantly but not completely restored renal function and structure in the ZDF rats [19].

In early stage of CKD, elevation of FGF23 represents an appropriate physiological response to prevent hyperphosphatemia. However, with CKD progression, the excess of biologically active FGF23 becomes no longer protective and may instead lead to pathological off-target effects [3]. Elevated circulating levels of FGF23 were associated with vascular dysfunction, atherosclerotic burden and left ventricular hyperthrophy in CKD patients [32], [33], and FGF23 directly induced hypertrophy of cultured cardiomyocytes [9]. Recently, inflammation has been identified as another potential off-target, in that higher FGF23 levels were independently associated with higher levels of inflammatory markers in patients with CKD [34]. Consistently, here, in ZDF rats the reduction of renal FGF23 production after treatment with ACE inhibitor was paralleled by less infiltrates of inflammatory cells in the renal interstitium. To maximize renoprotection in ZDF rats one could foresee adding other compounds to the ACE inhibitor. In the search for an effective therapy, molecules that have the ability to limit, but not to abrogate FGF23 production would represent a valuable approach. A recent study has shown vascular calcification associated with increased risk of mortality in CKD rats after FGF23 neutralization by chronic treatment with a FGF23 antibody [35], suggesting the importance of maintaining physiological levels of circulating FGF23.

Findings that in ZDF rats proteinuria preceded FGF23 upregulation in the kidney and that the antiproteinuric effect of ACE inhibitor was associated with reduced FGF23 expression, could be taken to suggest proteinuria as a potential trigger of renal FGF23. Future studies are needed to address this possibility. In this context, a recent study demonstrated that in patients with CKD FGF23 was positively associated with proteinuria [36]. Consistenly, we have documented here a positive correlation between renal FGF23 expression and proteinuria in diabetic rats.

In conclusion, our data indicate that in experimental type 2 diabetes 1. the kidney is a site of FGF23 production; 2. during the progression of the disease, renal FGF23 increased in the face of Klotho and NaPi-2a co-transporter reduction; 3. ACE inhibitor therapy besides exhibiting antiproteinuric and renoprotective actions, attenuated FGF23 renal production and preserved the expression of Klotho resulting in sustained improvement of phosphate homeostasis. These data may offer new clues to understand how to interfere with the delicate balance of FGF23 and phosphorus in diabetes with potential implications in clinics.

## Supporting Information

Figure S1 **FGF23 mRNA expression evaluated by RT-PCR in kidney tissue from lean rats, and ZDF rats treated with vehicle or ramipril from 4 months of age.** Spleen, a known source of FGF23, was obtained from a control rat.
(TIFF)
