# Supplementary figures and images for: Renal Expression of FGF23 in Progressive Renal Disease of Diabetes and the Effect of Ace Inhibitor
Source: PLoS One. 2013 Aug 14;8(8):e70775. doi: 10.1371/journal.pone.0070775 (PMC3743899; doi:10.1371/journal.pone.0070775)

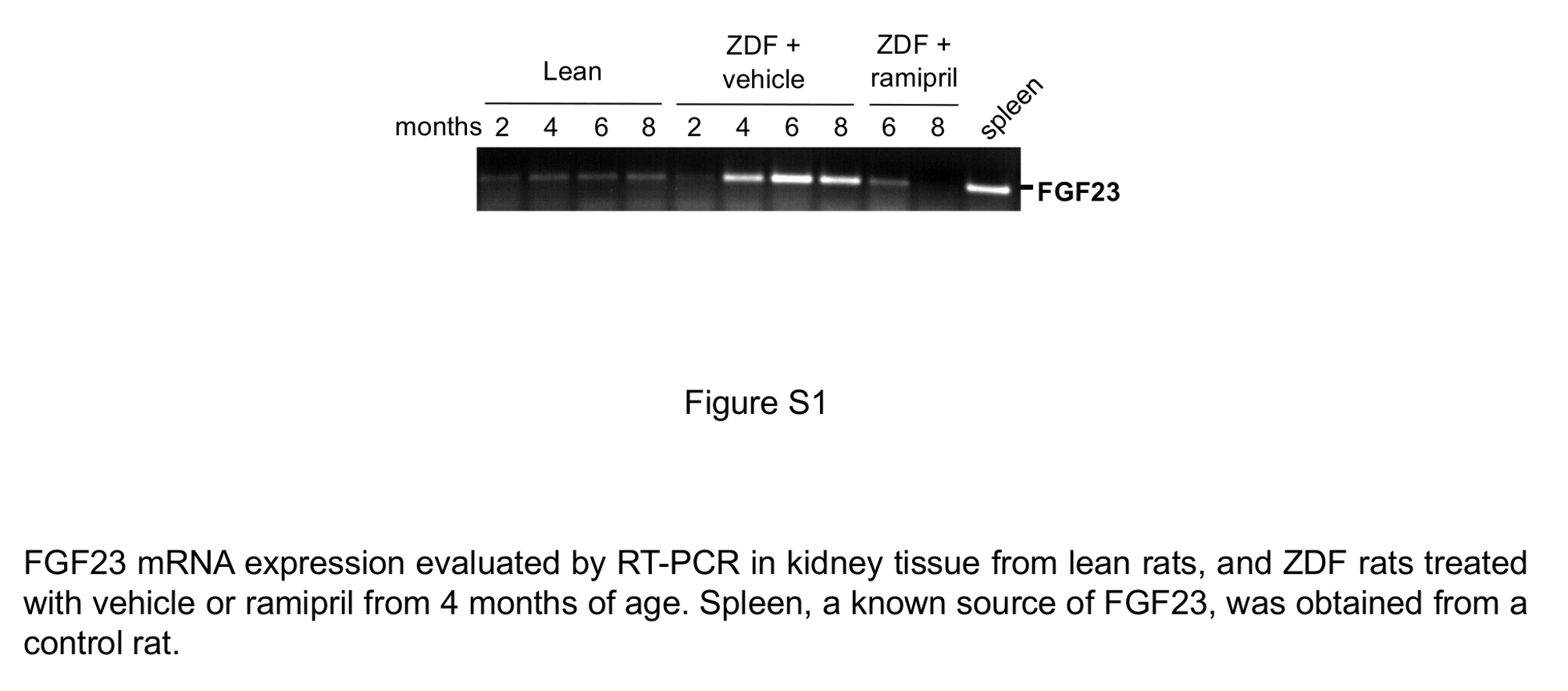

Supplement: Figure S1 — FGF23 mRNA expression evaluated by RT-PCR in kidney tissue from lean rats, and ZDF rats treated with vehicle or ramipril from 4 months of age. Spleen, a known source of FGF23, was obtained from a control rat. (TIFF) [file pone.0070775.s001.tiff]
